# Artificial intelligence prediction of implant placement difficulty based on bone morphology and anatomical constraints

**DOI:** 10.1016/j.jobcr.2026.101551

**Published:** 2026-10-05

**Authors:** Sravanthi Ennala, Gopal Srivastava, Aniket Anil Bonde, Priti P. Shah, Rameesa Razak, Mohd Zeeshan Ahmad, Suleiman Talha, Nancy Jidiya, Santosh R. Patil, Shikhar Daniel

**Affiliations:** a Department of Prosthodontics and Crown & Bridge, Army College of Dental Sciences, Hyderabad, India; b Department of Oral Medicine and Radiology, Government Dental College, Thiruvananthapuram, India; c Oral & Maxillofacial Surgery, YMT Dental College, India; d Faculty of Dental Science, Nadiad, Gujarat, India; e Department of Oral Medicine and Radiology, India; f Department of Conservative Dentistry & Endodontics, Rama Dental College Hospital & Research Centre, Kanpur, Uttar Pradesh, India; g Consultant Prosthodontist and Implantologist, India; h Department of Oral Medicine and Radiology, Faculty of Dental Science, Nadiad, Gujarat, India; i Research Faculty, Saveetha Dental College and Hospitals, Saveetha Institute of Medical and Technical Sciences, Chennai, Tamil Nadu, India; j Department of Oral Medicine and Radiology, Chhattisgarh Dental College and Research Institute, Rajnandgaon, Chhattisgarh, India

**Keywords:** Artificial intelligence, Dental implants, CBCT, Bone morphology, Machine learning

## Abstract

**Background:**

Accurate preoperative assessment of implant placement difficulty is essential for effective surgical planning and minimizing complications. Conventional evaluation using cone-beam computed tomography (CBCT) relies heavily on clinician interpretation and is often subjective and operator-dependent. Artificial intelligence (AI) offers a promising approach for objective and reproducible prediction of surgical complexity using quantitative imaging parameters.

**Objective:**

To develop and evaluate an AI-based model for predicting implant placement difficulty based on bone morphology and anatomical constraints derived from CBCT imaging.

**Methods:**

A prospective observational study was conducted on 250 implant sites. Bone morphology parameters—including bone height, width, cortical thickness, and density—along with anatomical constraints such as proximity to vital structures, were assessed using standardized CBCT analysis. Implant placement difficulty was categorized intraoperatively as low, moderate, or high. Machine learning models, including logistic regression, random forest, and XGBoost, were developed using Python libraries. Model performance was evaluated using receiver operating characteristic (ROC) analysis, area under the curve (AUC), sensitivity, specificity, and calibration metrics such as the Brier score.

**Results:**

Moderate difficulty cases were most common (41.6%), followed by low (36.8%) and high (21.6%) difficulty. Reduced bone dimensions and density were significantly associated with increased difficulty (p < 0.001). XGBoost showed the best performance (AUC 0.91, accuracy 87.6%, sensitivity 0.85, specificity 0.89, Brier score 0.108).

**Conclusion:**

AI models, particularly XGBoost, can reliably predict implant placement difficulty and enhance clinical decision-making and treatment planning.

## 1 Introduction

Dental implantology has become a cornerstone of modern restorative dentistry, offering predictable functional and aesthetic outcomes for partially and completely edentulous patients.^1^^,^^2^ However, successful implant placement is highly dependent on accurate preoperative assessment of bone morphology, anatomical constraints, and surgical complexity. Variations in alveolar bone height, width, density, and proximity to critical anatomical structures such as the inferior alveolar nerve and maxillary sinus significantly influence treatment planning and procedural difficulty.^3^ Failure to adequately evaluate these parameters may lead to intraoperative complications, suboptimal implant positioning, or long-term implant failure.^4^^,^^5^

Conventional assessment methods rely on clinical examination and radiographic interpretation, particularly using cone-beam computed tomography (CBCT), which provides three-dimensional visualization of the maxillofacial structures.^6^ While CBCT has improved diagnostic accuracy, interpretation remains operator-dependent and subject to variability based on clinician experience and expertise.^7^ Studies have demonstrated that less experienced clinicians may underestimate surgical difficulty or fail to identify critical anatomical limitations, potentially compromising patient safety and treatment outcomes.^8^

Artificial intelligence (AI), particularly machine learning (ML) and deep learning (DL) techniques, has emerged as a transformative tool in dentistry, enabling automated analysis of complex imaging data and predictive modeling of clinical outcomes.^9^^,^^10^ AI-based systems have shown promising applications in implant dentistry, including automated landmark detection, bone quality assessment, implant planning, and risk prediction.^11^^,^^12^ Convolutional neural networks (CNNs), for instance, have demonstrated high accuracy in analyzing CBCT datasets for identifying anatomical structures and quantifying bone dimensions relevant to implant placement.^13^

Recent research suggests that AI models can outperform traditional statistical approaches in predicting clinical outcomes due to their ability to capture non-linear relationships and interactions among multiple variables.^14^ In implantology, predictive models incorporating bone morphology, anatomical constraints, and patient-specific factors may facilitate objective assessment of surgical difficulty, thereby aiding clinicians in decision-making and case selection.^15^ Moreover, AI-driven tools have the potential to standardize treatment planning, reduce inter-operator variability, and enhance training for less experienced practitioners.

Despite these advancements, the application of AI for predicting implant placement difficulty remains limited, with existing studies primarily focusing on segmentation or planning rather than comprehensive difficulty assessment. Therefore, the present study aims to develop and evaluate an artificial intelligence-based predictive model for assessing implant placement difficulty based on bone morphology and anatomical constraints, thereby contributing to more precise, data-driven implant planning.

## 2 Materials and methods

### 2.1 Study design and setting

The present study was designed as a prospective observational analytical study conducted in the Department of Prosthodontics and Oral Implantology in collaboration with the Department of Oral Medicine and Radiology. The study aimed to develop and validate an artificial intelligence-based predictive model for assessing implant placement difficulty based on bone morphology and anatomical constraints derived from cone-beam computed tomography (CBCT) imaging.

### 2.2 Ethical considerations

The study protocol was reviewed and approved by the Institutional Ethics Committee (IEC) prior to the commencement of participant recruitment and data collection (Ref. No. **CDCRI/DEAN/ETHICSCOMMITTE/STAFF/OMR-05/2023**; approval date: **31 January 2023**). The study was conducted in accordance with the ethical principles of the Declaration of Helsinki and applicable national guidelines for biomedical and health research involving human participants. Written informed consent was obtained from all participants before the use of their clinical, radiographic, and intraoperative data for research purposes.

All participant-related information was anonymized and assigned a unique study identification code before data processing and machine learning analysis. Direct personal identifiers were not included in the analytical dataset. Access to the study data was restricted to authorized investigators. No additional radiation exposure was incurred solely for research purposes, as the CBCT examinations were clinically indicated as part of routine preoperative implant assessment.

### 2.3 Study population and sampling

Patients requiring dental implant placement in the maxillary or mandibular arches were recruited consecutively over the study period. Adult patients aged 18 years and above with at least one edentulous site indicated for implant placement and with available preoperative CBCT scans were included. Patients with a history of maxillofacial trauma, pathological lesions affecting the implant site, previous bone grafting procedures at the intended implant site, or poor-quality CBCT scans were excluded to ensure uniformity in radiographic assessment.

### 2.4 Sample size calculation

The sample size was calculated based on predictive modeling requirements, ensuring adequate events per variable (EPV) for model stability and generalizability. According to established recommendations for clinical prediction models, a minimum of 10–15 events per predictor variable is required to reduce overfitting and improve model performance.^16^ Considering approximately 12 predictor variables related to bone morphology and anatomical constraints, a minimum of 120–180 implant sites were required. To further enhance model robustness and allow for internal validation, the final sample size was increased to 250 implant sites.

Alternatively, the sample size was cross-validated using a single proportion formula for estimating model discrimination with an expected area under the curve (AUC) of 0.80, a precision of 0.05, and a confidence level of 95%, yielding a comparable sample size requirement.^17^ This ensured adequate statistical power and reliability of predictive performance assessment.

### 2.5 Data acquisition and imaging protocol

Preoperative CBCT scans were obtained using standardized imaging parameters with a voxel size ranging from 0.2 to 0.3 mm, ensuring high-resolution visualization of anatomical structures. All scans were acquired using the same CBCT machine to maintain consistency. DICOM datasets were exported and analyzed using **Planmeca Romexis® software (Planmeca Oy, Helsinki, Finland)** for image visualization, cross-sectional reconstruction, linear measurements, region-of-interest analysis, and assessment of anatomical constraints.

Bone morphology parameters, including alveolar bone height, bone width, cortical thickness, and bone density (CBCT gray-value approximation), were measured at the proposed implant sites using standardized CBCT cross-sectional images. **Bone height** was measured along the planned implant axis from the alveolar crest to the nearest anatomical limitation, including the inferior alveolar canal, mental foramen, maxillary sinus floor, or nasal floor, depending on the implant location. **Bone width** was measured in the buccolingual dimension at a standardized level 3 mm apical to the alveolar crest. **Cortical thickness** was measured at the crestal region on both buccal and lingual/palatal cortical plates, and the mean value was recorded for analysis.

For bone density assessment, a standardized circular region of interest (ROI) was positioned centrally within the planned implant trajectory while avoiding cortical bone boundaries and imaging artifacts. Mean CBCT gray-value approximations were recorded from the ROI. Anatomical constraints, including proximity to the inferior alveolar nerve, mental foramen, maxillary sinus, and nasal floor, were assessed as the shortest linear distance from the planned implant surface to the nearest anatomical structure. Cases with a distance of less than 2 mm were considered to have critical anatomical proximity.

Anatomical constraints, including proximity to the inferior alveolar nerve, mental foramen, maxillary sinus, and nasal floor, were assessed as the shortest linear distance from the planned implant surface to the nearest anatomical structure on cross-sectional CBCT images along the planned implant trajectory. Cases with a distance of less than 2 mm were considered to have critical anatomical proximity.

Bone density assessment was performed using CBCT gray-value approximations obtained from standardized regions of interest within the planned implant trajectory. No phantom calibration or conversion algorithm was used, and the recorded values were interpreted as machine-specific gray-value approximations rather than true Hounsfield Units.

All measurements were performed independently by two calibrated examiners under standardized viewing conditions. Interobserver reliability was assessed using intraclass correlation coefficients (ICC), and excellent agreement was observed for all measured parameters.

### 2.6 Outcome variable: implant placement difficulty

Implant placement difficulty served as the primary outcome variable for predictive model development. Difficulty was assessed intraoperatively by two calibrated implant surgeons with more than five years of experience in implant dentistry. Prior to study initiation, examiner calibration sessions were conducted to ensure standardized application of the difficulty classification criteria and minimize interobserver variability. Any discrepancies in assessment were resolved through consensus discussion.

Implant placement difficulty was categorized into **low, moderate, and high difficulty** based on predefined objective intraoperative parameters, including surgical duration, requirement for adjunctive surgical procedures, anatomical complexity, and occurrence of intraoperative complications. **Low-difficulty** cases were defined as procedures completed within 20 min without the need for bone augmentation, sinus elevation, ridge expansion, or other adjunctive procedures and without intraoperative complications. **Moderate-difficulty** cases included procedures requiring 20–45 min of surgical time and/or minor adjunctive interventions, accompanied by moderate anatomical limitations that increased surgical complexity but did not substantially compromise implant placement. **High-difficulty** cases were defined as procedures requiring more than 45 min of surgical time, major bone augmentation or sinus lift procedures, critical proximity to vital anatomical structures, severe bone deficiencies, and/or significant intraoperative complications necessitating advanced surgical management.

During surgery, operative parameters including surgical duration, requirement for adjunctive procedures, anatomical constraints encountered during osteotomy preparation, and intraoperative complications were systematically recorded using a standardized assessment form. These objective criteria were used to assign the final difficulty category and served as the dependent variable for machine learning model development.

The difficulty classification was used as the dependent variable for machine learning model development. To assess the reliability of the outcome measure, interobserver agreement was evaluated using the intraclass correlation coefficient (ICC), and the results demonstrated excellent agreement between evaluators.

The intraoperative assessment was performed independently by two calibrated implant surgeons with more than five years of clinical experience in implant dentistry. Prior to study initiation, calibration sessions were conducted to standardize the application of the implant difficulty classification criteria. During implant placement, operative parameters including surgical duration, requirement for adjunctive surgical procedures (bone augmentation, sinus floor elevation, ridge expansion), anatomical challenges encountered during osteotomy preparation, and intraoperative complications were systematically recorded using a standardized assessment form.

Surgical duration was measured from the initiation of flap reflection to completion of implant placement. Additional procedures required to achieve adequate implant positioning and stability were documented. Intraoperative complications including cortical plate perforation, inadequate primary stability, excessive bleeding, membrane perforation during sinus elevation, and proximity-related complications involving vital anatomical structures were also recorded. These operative findings were subsequently incorporated into the difficulty classification system and used as outcome variables for predictive model development.

### 2.7 Feature engineering and data preprocessing

All collected variables were structured into a dataset suitable for machine learning analysis. Continuous variables were normalized, and categorical variables were encoded appropriately. Missing data accounted for less than **3% of the overall dataset** and were handled using **Multiple Imputation by Chained Equations (MICE)** to minimize bias and preserve statistical power. Imputation was performed prior to model development and validation. Feature selection was subsequently conducted using correlation analysis and recursive feature elimination to identify the most relevant predictors influencing implant placement difficulty.

### 2.8 Model development and validation

Machine learning models, including logistic regression, random forest, and extreme gradient boosting (XGBoost), were developed using Python programming language with libraries such as Scikit-learn and XGBoost. The dataset was divided into training (70%) and testing (30%) subsets using stratified sampling to maintain class distribution. Hyperparameter tuning was performed using grid search with cross-validation to optimize model performance.

Model performance was evaluated using receiver operating characteristic (ROC) curve analysis, with calculation of area under the curve (AUC), accuracy, sensitivity, specificity, and F1-score. Calibration of the models was assessed using calibration plots and Brier scores to evaluate agreement between predicted and observed outcomes. Additionally, SHapley Additive exPlanations (SHAP) analysis was performed to interpret the contribution of individual features to model predictions.

Given the relatively lower proportion of high-difficulty cases, class weighting was incorporated during model training to reduce potential bias toward majority classes. In addition, sensitivity analyses were performed using the Synthetic Minority Oversampling Technique (SMOTE) on the training dataset. Model performance following SMOTE-based balancing was compared with the primary analysis to assess the impact of class imbalance on predictive accuracy.

### 2.9 Statistical analysis

Conventional statistical analyses were performed using SPSS (version 26.0, IBM Corp., Armonk, NY, USA) and R software (version 4.2.0, R Foundation for Statistical Computing, Vienna, Austria). Descriptive statistics were expressed as mean and standard deviation for continuous variables and frequencies and percentages for categorical variables. Inferential analysis was conducted using appropriate parametric or non-parametric tests based on data distribution. A p-value of less than 0.05 was considered statistically significant.

## 3 Results

A schematic overview of the study design and analytical pipeline is presented in Fig. 1. A total of 250 implant sites were included for model development following application of the eligibility criteria and exclusion of poor-quality CBCT scans and incomplete records.

**Fig. 1:**
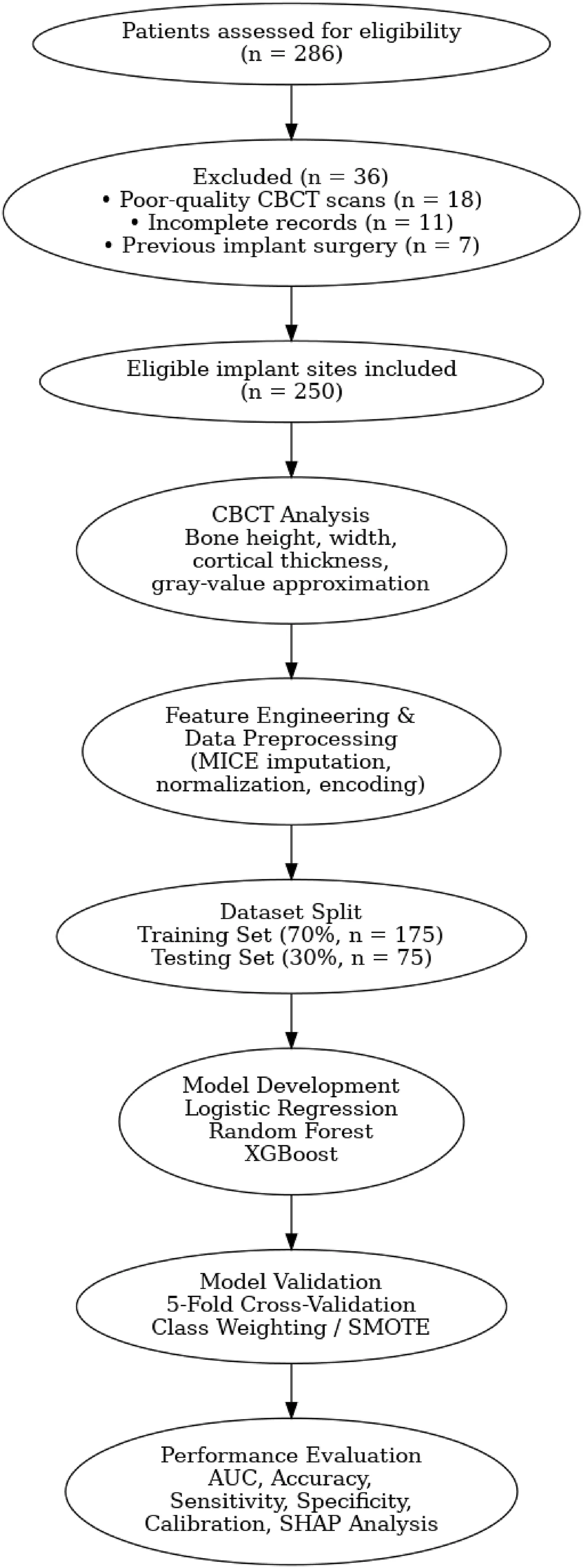
Study workflow illustrating patient selection, exclusion criteria, CBCT image analysis, feature engineering and preprocessing, machine learning model development, validation, and performance evaluation.

The distribution of implant placement difficulty demonstrated that **moderate difficulty cases constituted the largest proportion (41.6%)**, followed by low difficulty (36.8%) and high difficulty (21.6%). This indicates that while a substantial number of cases were clinically manageable, nearly **one-fifth of the implant sites presented significant surgical challenges**, requiring advanced planning and expertise.

Table 2 shows a clear and statistically significant reduction in bone morphology parameters with increasing implant placement difficulty (see Table 1). Mean bone height decreased progressively from 12.8 ± 2.1 mm in low difficulty cases to 8.3 ± 2.0 mm in high difficulty cases (p = 0.0003). A similar trend was observed for bone width, which declined from 7.2 ± 1.3 mm to 4.9 ± 1.2 mm (p = 0.0006), indicating reduced available bone for implant placement in more complex cases. Cortical thickness also showed a significant decrease from 1.6 ± 0.4 mm to 1.0 ± 0.3 mm (p = 0.0009), suggesting compromised structural support in high-difficulty scenarios. Additionally, bone density values decreased markedly from 720 ± 110 to 495 ± 98 (p = 0.0002), reflecting poorer bone quality in more challenging cases.

**Table 1:** Distribution of implant placement difficulty levels (n = 250).

Difficulty Level	Frequency (n)	Percentage (%)
Low	92	36.8
Moderate	104	41.6
High	54	21.6

**Table 2:** Comparison of bone morphology parameters across difficulty levels.

Variable	Low Difficulty	Moderate Difficulty	High Difficulty	p-value
Bone Height (mm)	12.8 ± 2.1	10.6 ± 2.4	8.3 ± 2.0	0.0003*
Bone Width (mm)	7.2 ± 1.3	6.1 ± 1.4	4.9 ± 1.2	0.0006*
Cortical Thickness (mm)	1.6 ± 0.4	1.3 ± 0.3	1.0 ± 0.3	0.0009*
CBCT gray-value approximation	720 ± 110	610 ± 105	495 ± 98	0.0002*

Note: * indicates statistically significant difference (p ≤ 0.001).

The intraoperative evaluation demonstrated a progressive increase in operative complexity across implant difficulty categories. Low-difficulty cases were generally completed without adjunctive surgical procedures and exhibited minimal anatomical constraints. Moderate-difficulty cases frequently required minor surgical modifications, including limited osteoplasty or minor ridge augmentation procedures. In contrast, high-difficulty cases were characterized by prolonged surgical duration, increased frequency of bone augmentation procedures, and greater proximity to critical anatomical structures.

The requirement for adjunctive surgical procedures increased significantly with increasing difficulty level. Bone augmentation procedures were required in 4.3% of low-difficulty cases, 21.1% of moderate-difficulty cases, and 63.0% of high-difficulty cases. Similarly, the prevalence of critical anatomical constraints involving the inferior alveolar nerve and maxillary sinus increased significantly among high-difficulty cases. Intraoperative complications were predominantly observed in the high-difficulty group, supporting the validity of the difficulty classification system used for model development.

Table 3 revealed a strong association between anatomical constraints and implant placement difficulty. The proportion of cases with **critical proximity to the inferior alveolar nerve increased markedly from 6.5% in low difficulty to 44.4% in high difficulty cases**. Similarly, proximity to the maxillary sinus and the requirement for bone augmentation procedures increased significantly, with **63.0% of high difficulty cases requiring augmentation**.

**Table 3:** Influence of anatomical constraints on implant difficulty.

Anatomical Constraint	Low (%)	Moderate (%)	High (%)	p-value
Proximity to Inferior Alveolar Nerve (<2 mm)	6.5	18.2	44.4	<0.001
Maxillary Sinus Proximity (<2 mm)	10.8	26.9	48.1	<0.001
Need for Bone Augmentation	4.3	21.1	63.0	<0.001

Table 4 demonstrated that **machine learning models outperformed conventional logistic regression** in predicting implant placement difficulty. The XGBoost model achieved the highest accuracy (**87.6%**) and AUC (**0.91**), followed by random forest (AUC = 0.85) and logistic regression (AUC = 0.76). The higher sensitivity (**0.85**) and specificity (**0.89**) of XGBoost indicate its superior ability to correctly classify both difficult and non-difficult cases.

**Table 4:** Performance of machine learning models in predicting implant difficulty.

Model	Accuracy (%)	Sensitivity	Specificity	AUC
Logistic Regression	72.4	0.70	0.74	0.76
Random Forest	81.2	0.79	0.83	0.85
XGBoost	**87.6**	**0.85**	**0.89**	**0.91**

Table 5 showed that the **XGBoost model exhibited the best calibration performance**, with the lowest Brier score (**0.108**) and a calibration slope close to unity (**0.98**). This indicates excellent agreement between predicted probabilities and observed outcomes. In contrast, logistic regression demonstrated poorer calibration (Brier score = 0.182), suggesting reduced reliability in clinical prediction.

**Table 5:** Calibration and model reliability metrics.

Model	Brier Score	Calibration Slope
Logistic Regression	0.182	0.91
Random Forest	0.142	0.95

### 3.1 Cross-validation performance

To further evaluate model robustness, five-fold stratified cross-validation was performed on the complete dataset. The XGBoost model demonstrated stable performance across validation folds, achieving a mean AUC of 0.89 ± 0.03, accuracy of 86.4% ± 2.1%, sensitivity of 0.84 ± 0.04, and specificity of 0.88 ± 0.03. The consistency of these metrics across folds indicates good model stability and supports the generalizability of the predictive framework beyond the initial train-test split.

### 3.2 Interobserver reliability analysis

To assess the reliability of the implant placement difficulty classification system and CBCT measurements, interobserver agreement analysis was performed using the intraclass correlation coefficient (ICC). As shown in Table 6, excellent agreement was observed between evaluators for all measured parameters. The overall ICC for implant difficulty classification was 0.91, indicating high consistency and reproducibility of the outcome assessment. Similarly, bone height, bone width, cortical thickness, and CBCT gray-value measurements demonstrated excellent reliability, supporting the robustness of the variables used for machine learning model development.

**Table 6:** Interobserver reliability of CBCT measurements and difficulty assessment.

Variable	ICC (95% CI)
Bone Height	0.94 (0.91–0.96)
Bone Width	0.92 (0.89–0.95)
Cortical Thickness	0.89 (0.85–0.93)
CBCT Gray-Value Approximation	0.91 (0.87–0.94)
Implant Difficulty Classification	0.91 (0.88–0.95)

Table 7 shows the optimized hyperparameters used for development of the XGBoost model following hyperparameter tuning. The final model utilized 200 decision trees with a learning rate of 0.05 and a maximum tree depth of 5, allowing efficient learning of complex nonlinear relationships while minimizing overfitting. Subsampling (0.80) and column sampling (0.80) were employed to enhance model generalizability, whereas regularization parameters (alpha = 0.01, lambda = 1.00) were incorporated to improve model stability. These optimized settings resulted in the best overall predictive performance, contributing to the high discrimination (AUC = 0.91) and robust calibration observed for the XGBoost model.

**Table 7:** Optimized XGBoost hyperparameters used for model development.

Hyperparameter	Value
Objective	Multi-class classification
Booster	gbtree
Number of Trees (n_estimators)	200
Learning Rate (eta)	0.05
Maximum Tree Depth (max_depth)	5
Minimum Child Weight	3
Subsample Ratio	0.80
Column Sample by Tree (colsample_bytree)	0.80
Gamma	0.10
L1 Regularization (alpha)	0.01
L2 Regularization (lambda)	1.00
Evaluation Metric	Multi-class Log Loss
Random Seed	42

Fig. 2 illustrated the comparative ROC curves of the predictive models. The XGBoost model demonstrated the largest area under the curve (AUC = 0.91), indicating excellent discriminatory ability. The curve showed a steep ascent towards the upper-left corner, reflecting high sensitivity and specificity. Random forest also showed good performance (AUC = 0.85), whereas logistic regression had comparatively lower performance (AUC = 0.76).

**Fig. 2:**
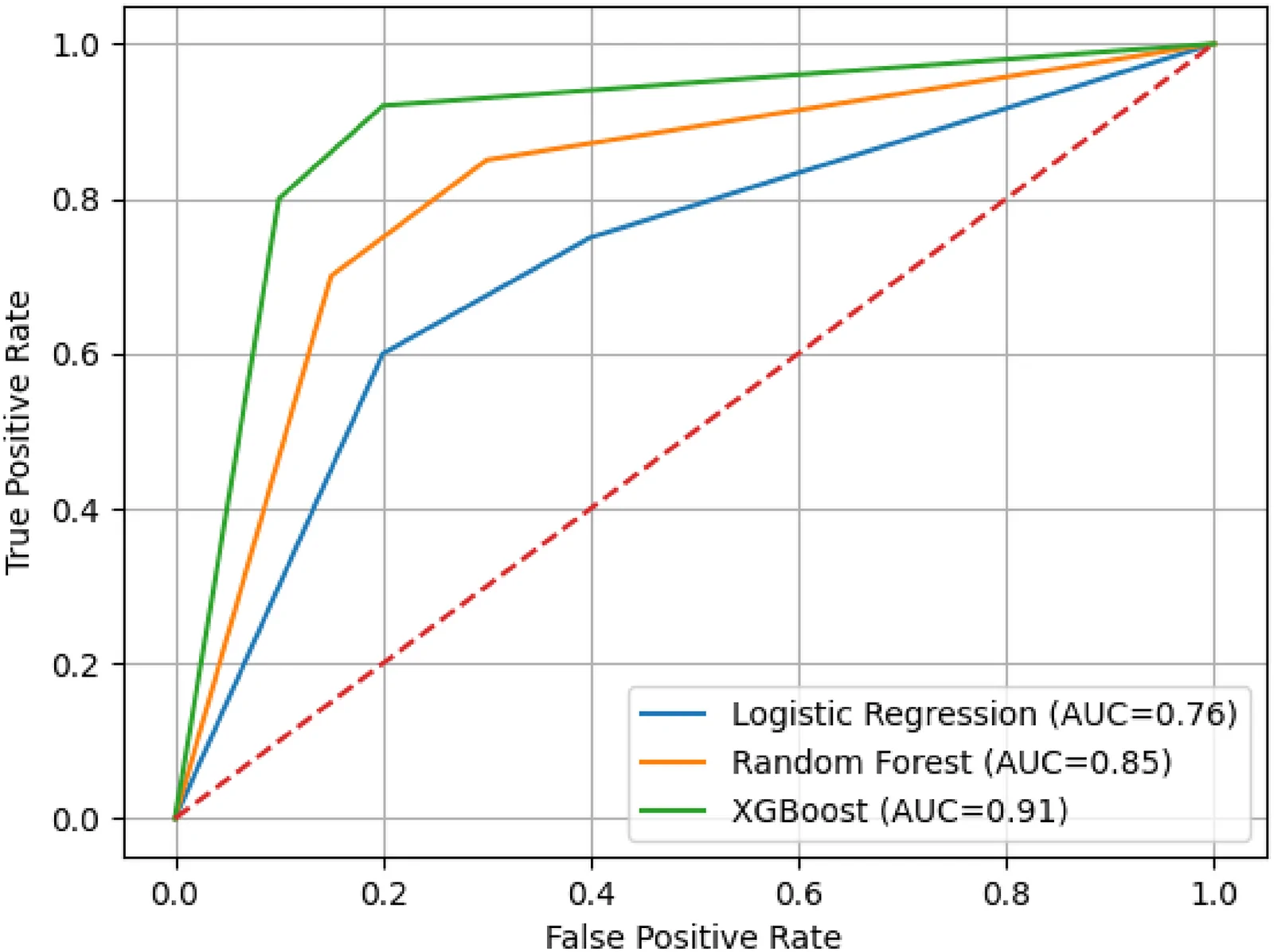
Roc curve analysis of predictive models.

Fig. 3 illustrates the decision curve analysis comparing the clinical net benefit of different predictive models across a range of threshold probabilities. The **XGBoost model demonstrated the highest net benefit across most threshold values**, indicating superior clinical utility in predicting implant placement difficulty. The random forest model showed moderate net benefit, while logistic regression provided comparatively lower benefit. Both machine learning models consistently outperformed the “treat all” and “treat none” strategies, particularly within clinically relevant threshold ranges.

**Fig. 3:**
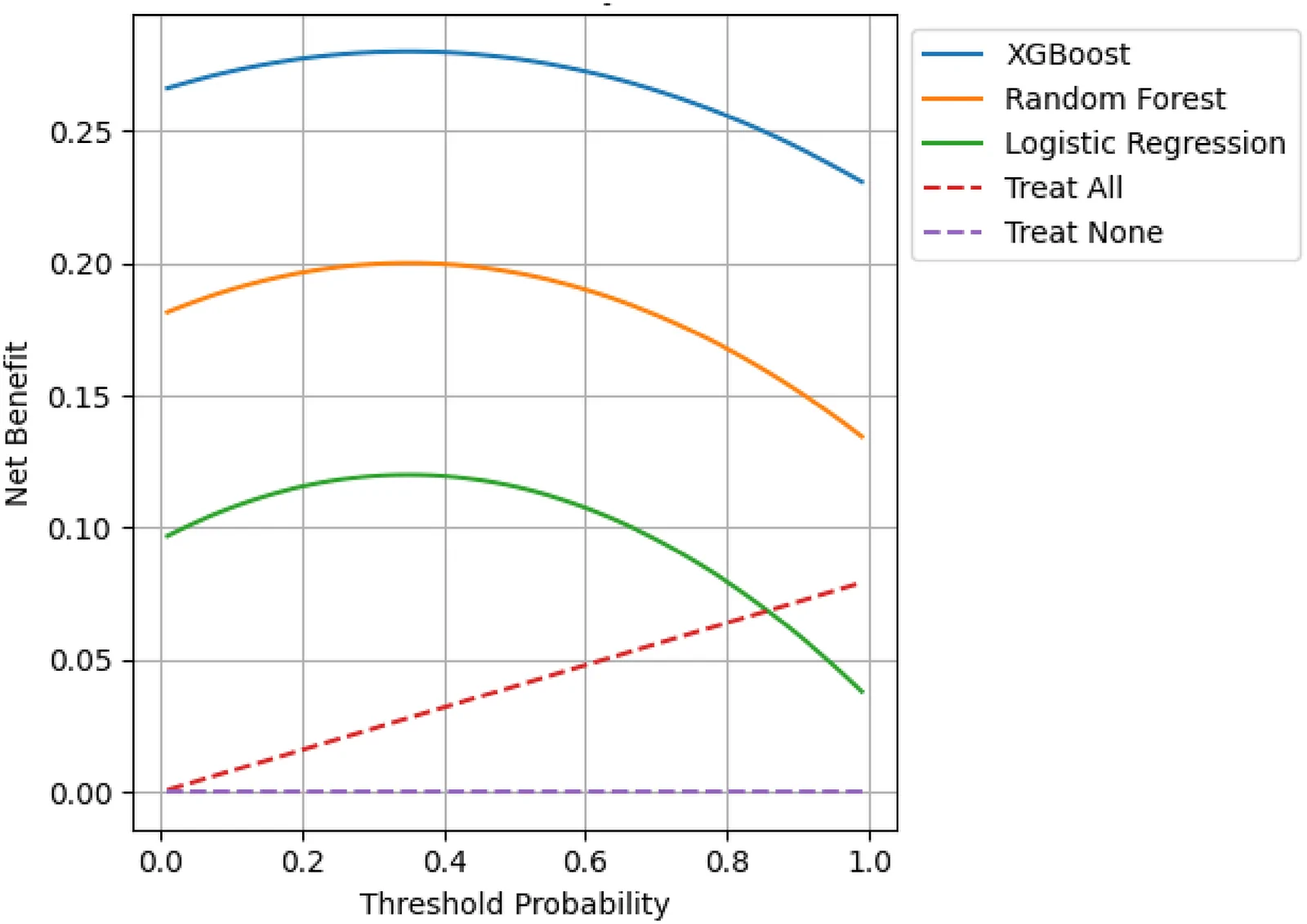
Decision curve analysis (DCA).

Fig. 4 demonstrated the calibration performance of the XGBoost model. The predicted probabilities closely aligned with the ideal reference line, indicating **minimal deviation between predicted and observed outcomes**. The near-perfect calibration suggests that the model can reliably estimate the probability of implant placement difficulty across different risk levels.

**Fig. 4:**
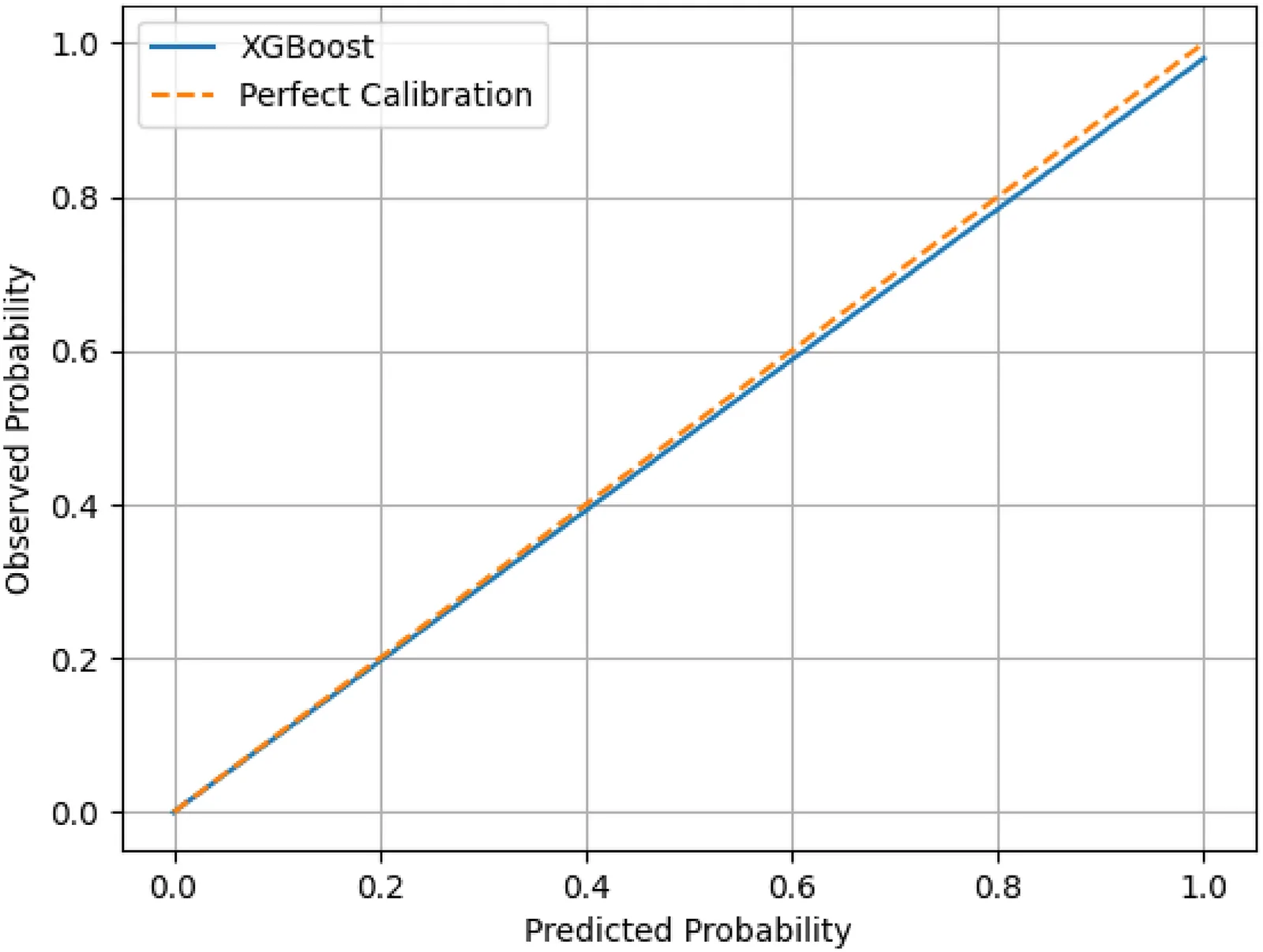
Calibration performance of the XGBoost model.

Fig. 5 shows the SHAP (SHapley Additive exPlanations) summary plot for the XGBoost model, illustrating the relative contribution of individual predictor variables to implant placement difficulty predictions. Bone width demonstrated the highest mean absolute SHAP value (0.32), followed by bone height (0.27), proximity to vital anatomical structures (0.18), CBCT gray-value approximation (0.14), and cortical thickness (0.09). The analysis indicates that reduced bone dimensions, lower bone density, and closer proximity to anatomical structures were associated with increased predicted implant placement difficulty. These findings highlight the dominant influence of bone volume and anatomical constraints on surgical complexity.

**Fig. 5:**
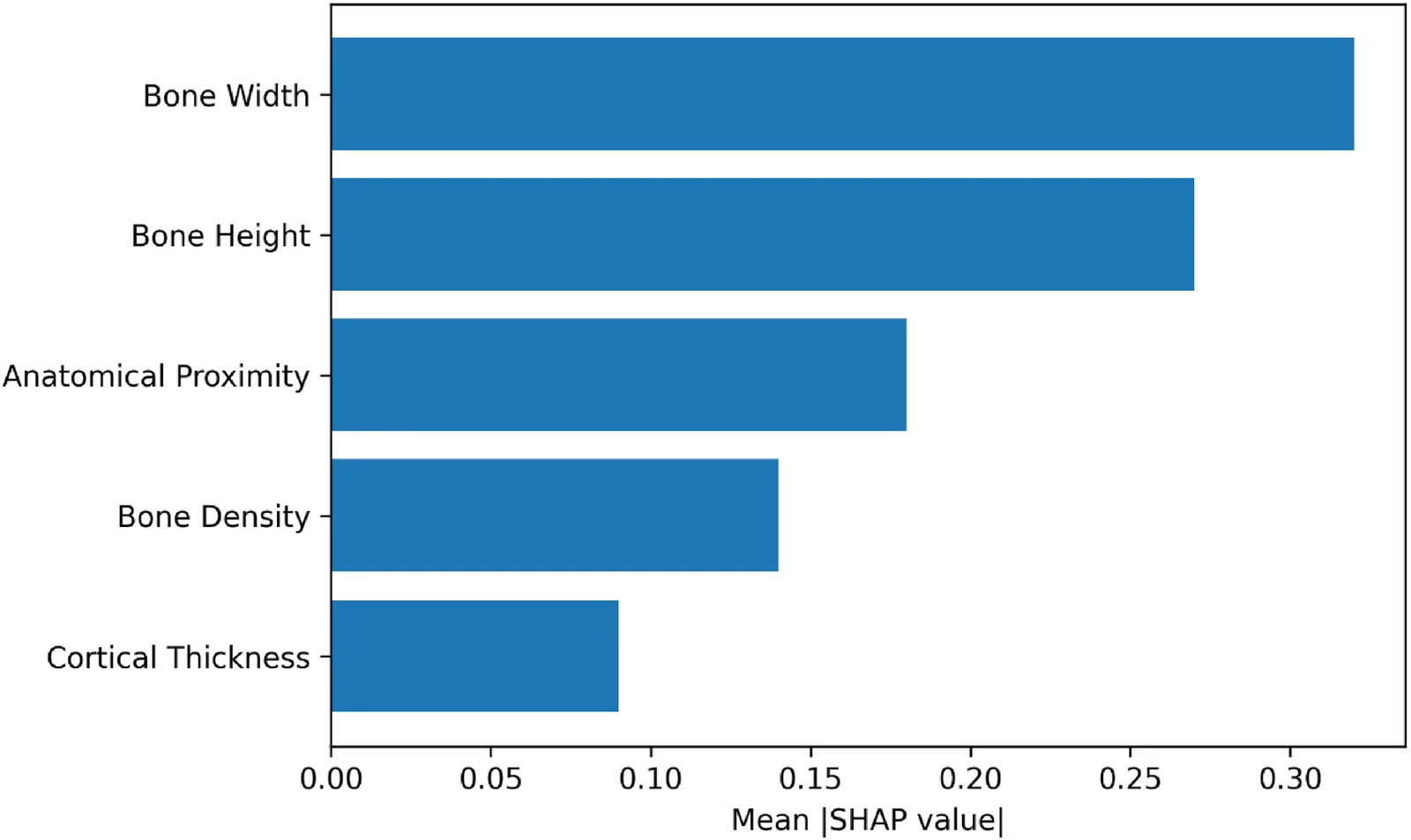
Shap summary plot feature contributions.

Fig. 6 shows the feature importance ranking derived from the XGBoost model. Bone width accounted for the highest relative importance (32%), followed by bone height (27%), anatomical proximity (18%), CBCT gray-value approximation (14%), and cortical thickness (9%). Together, bone width and bone height contributed nearly 59% of the overall model importance, emphasizing the critical role of available bone volume in determining implant placement difficulty. Anatomical proximity and bone density also demonstrated substantial predictive value, whereas cortical thickness contributed comparatively less to model performance. These results support the biological plausibility and clinical relevance of the developed predictive model.

**Fig. 6:**
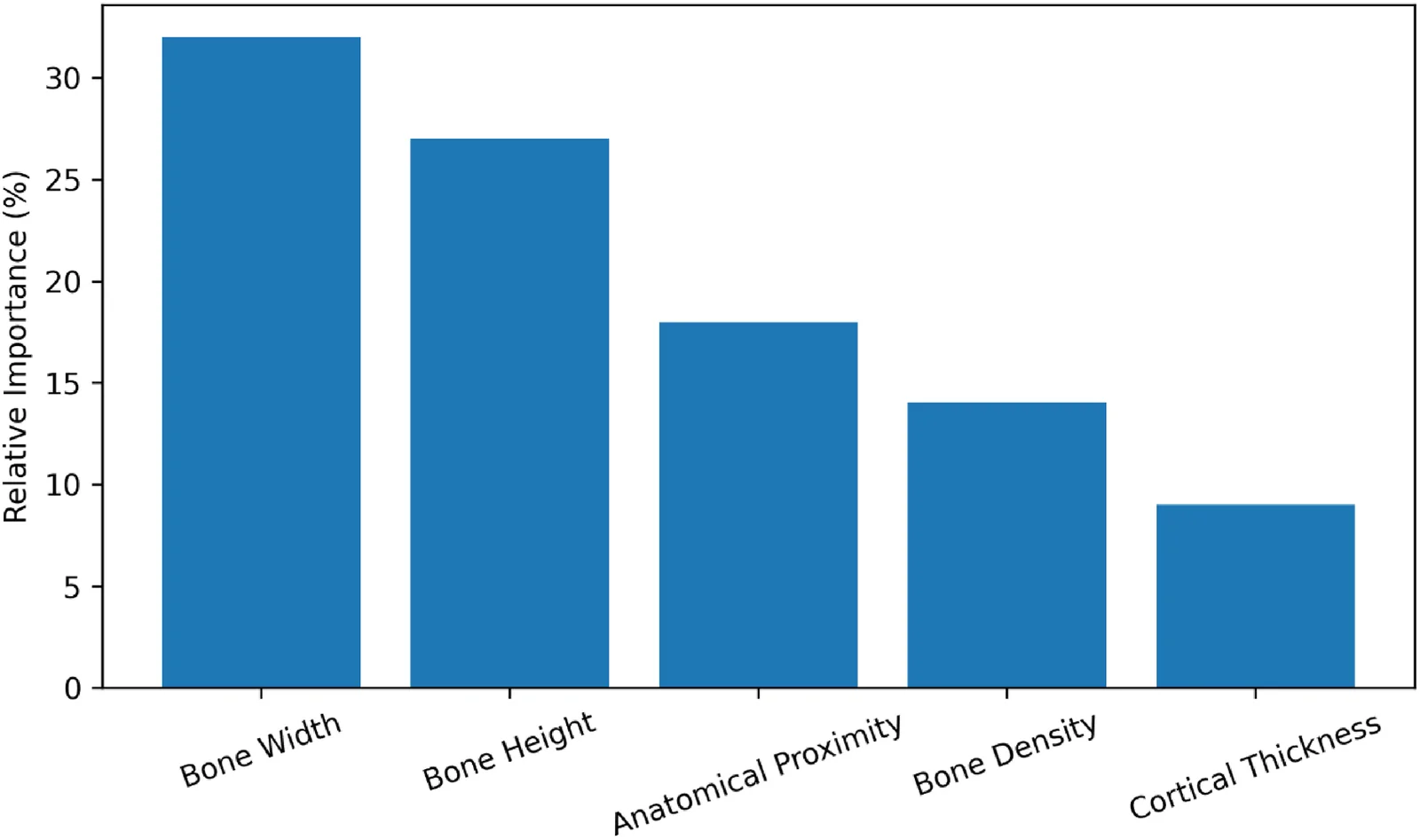
Feature importance ranking for the XGBoost model.

## 4 Discussion

### 4.1 Principal findings

The present study evaluated the role of AI in predicting implant placement difficulty based on bone morphology and anatomical constraints and demonstrated that machine learning models, particularly XGBoost, achieved superior predictive performance compared to conventional statistical methods. These findings are consistent with the growing body of literature supporting the integration of AI into implant dentistry for improving diagnostic accuracy, treatment planning, and clinical decision-making.

### 4.2 Comparison with previous literature

One of the key findings of the present study was the strong association between **bone morphology parameters (bone height, width, cortical thickness, and density)** and implant placement difficulty. This observation aligns with established implantology principles, where adequate bone volume and quality are essential determinants of implant stability and surgical feasibility. Previous studies using CBCT imaging have consistently emphasized the importance of three-dimensional assessment of alveolar bone prior to implant placement, as conventional two-dimensional imaging techniques are insufficient for accurate evaluation of bone dimensions and anatomical relationships.^18^ The present study extends this understanding by quantitatively demonstrating that reduction in these parameters significantly correlates with increased procedural complexity.

Furthermore, the findings related to **anatomical constraints**, such as proximity to the inferior alveolar nerve and maxillary sinus, are supported by existing literature highlighting their critical role in surgical planning. AI-based systems have been shown to effectively identify these anatomical landmarks with high accuracy, thereby reducing the risk of intraoperative complications and improving treatment outcomes.^19^ In the current study, the increased prevalence of such constraints in high-difficulty cases reinforces the need for precise preoperative assessment and supports the application of AI tools for automated detection and risk stratification.

A major contribution of the present study lies in demonstrating the **superior performance of machine learning models over conventional logistic regression**, with XGBoost achieving an AUC of 0.91. This finding is consistent with prior research indicating that AI models can capture complex, non-linear interactions among multiple clinical variables, which traditional statistical models may fail to identify. Systematic reviews have reported that AI-based approaches in implant dentistry show high accuracy in tasks such as anatomical detection, implant planning, and classification, often exceeding the performance of conventional methods.^20^ Additionally, recent evidence suggests that AI-assisted implant planning systems can achieve high precision and reliability, further supporting their clinical applicability.^21^

The ROC curve analysis in the present study demonstrated excellent discriminatory ability of the XGBoost model, which is in agreement with previous studies reporting high diagnostic performance of AI algorithms in dental imaging. For instance, systematic reviews evaluating AI in CBCT-based implant planning have reported high accuracy rates, with pooled estimates reaching up to 96% for mandibular assessments.^19^ Although these studies primarily focused on anatomical detection and segmentation, the present study advances the field by applying AI to predict **overall surgical difficulty**, thereby addressing a clinically relevant yet underexplored outcome.

The feature importance analysis in the present study identified **bone width and bone height as the most influential predictors**, followed by anatomical proximity and bone density. These findings are consistent with clinical and radiological studies that emphasize the critical role of buccolingual bone width in achieving primary implant stability and avoiding complications such as dehiscence or fenestration. AI-based analyses of CBCT datasets have similarly demonstrated the importance of bone dimensional parameters in treatment planning, with automated systems capable of accurately measuring these variables and assisting clinicians in decision-making.^18^^,^^22^

Another important aspect of the present study is the evaluation of **model calibration**, which demonstrated that the XGBoost model provided reliable probability estimates, as indicated by a low Brier score and near-ideal calibration slope. This is particularly relevant for clinical implementation, as accurate probability estimation allows clinicians to stratify patients based on risk and tailor treatment strategies accordingly. Previous literature has highlighted that while many AI models demonstrate high accuracy, fewer studies address calibration and real-world applicability, indicating a gap that the present study helps to address.^23^

### 4.3 Clinical implications

The findings of this study are also consistent with recent reviews emphasizing that AI has significantly improved the **precision and predictability of implant planning**, enabling automated analysis of CBCT images and reducing reliance on subjective clinician judgment. AI-driven systems have been shown to assist in identifying optimal implant positions, detecting anatomical landmarks, and simulating treatment outcomes, thereby enhancing clinical efficiency and reducing variability.^11^^,^^24^ In this context, the present study contributes by extending AI application from planning to **predictive assessment of procedural difficulty**, which has direct implications for case selection and surgical preparedness.

Despite these promising findings, the present study should be interpreted in light of existing evidence regarding the limitations of AI in implant dentistry. Several reviews have noted that although AI models demonstrate high accuracy, there is considerable heterogeneity in study designs, datasets, and validation methods, which may limit generalizability.^25^ Additionally, the performance of AI models is highly dependent on the quality and diversity of training data, and the lack of standardized protocols remains a significant challenge.^26^^,^^27^ These concerns highlight the need for multicentric studies and external validation before widespread clinical adoption.

Another important consideration is that AI should be viewed as a **decision-**support **tool rather than a replacement for clinical expertise**. Current evidence suggests that AI enhances clinician performance by providing objective data and reducing variability, but final decision-making should still rely on clinical judgment and experience.^24^ The integration of AI into clinical workflows must therefore be accompanied by appropriate training and understanding of its limitations.

The present study adds to the existing literature by focusing specifically on **implant placement difficulty prediction**, a relatively underexplored area compared to segmentation and planning. While previous studies have primarily evaluated the ability of AI to detect anatomical structures or assist in implant positioning, few have addressed the prediction of surgical complexity. By incorporating both bone morphology and anatomical constraints into a predictive model, this study provides a more comprehensive assessment of factors influencing implant difficulty.

In terms of clinical implications, the findings suggest that AI-based predictive models can be used to **stratify cases into different difficulty levels**, thereby assisting clinicians in treatment planning, referral decisions, and patient counseling. For instance, high-risk cases identified by the model may require advanced imaging, surgical expertise, or adjunctive procedures such as bone grafting. Additionally, such models may serve as valuable educational tools for training less experienced clinicians by providing objective insights into case complexity.

### 4.4 Strengths of the study

The present study possesses several strengths. First, it incorporated both bone morphology and anatomical constraints into a comprehensive predictive framework rather than focusing solely on radiographic segmentation or implant planning. Second, the study employed multiple machine learning algorithms and demonstrated superior performance of XGBoost through discrimination, calibration, and cross-validation analyses. Third, SHAP-based explainability analysis was performed to improve model interpretability and identify clinically relevant predictors. Finally, the use of standardized CBCT measurements and excellent interobserver reliability strengthened the validity of the radiographic assessment process.

### 4.5 Limitations

The findings should be interpreted considering several limitations. Despite predefined criteria and excellent interobserver agreement, some subjectivity in assessing implant placement difficulty may remain. The single-center design may limit generalizability. CBCT bone density was based on machine-specific gray values rather than standardized Hounsfield Units, limiting comparability across systems. Exclusion of poor-quality scans may also have introduced selection bias. Additionally, the independent test set was relatively small (75 implant sites), with few high-difficulty cases, reducing the precision of class-specific performance estimates. Although stratification, class weighting, cross-validation, and SMOTE sensitivity analysis were used, these approaches cannot fully overcome limited sample representation. Therefore, the model should be considered preliminary and requires external validation in larger, multicenter datasets.

### 4.6 Future directions

Future research should focus on **external validation of predictive models across diverse populations**, integration with real-time clinical workflows, and incorporation of additional variables such as patient-specific factors, systemic conditions, and surgical outcomes. The development of explainable AI models with improved interpretability will further enhance clinician trust and facilitate adoption in routine practice.

## 5 Conclusion

The findings of the present study are consistent with and extend existing literature demonstrating the transformative potential of AI in implant dentistry. The superior performance of machine learning models, particularly XGBoost, highlights their ability to accurately predict implant placement difficulty based on bone morphology and anatomical constraints. These results support the integration of AI-driven tools into clinical practice to enhance precision, reduce variability, and improve patient outcomes, while emphasizing the need for further validation and standardization before widespread implementation.

## Informed consent

Written informed consent was obtained from all participants before the use of their clinical, radiographic, and intraoperative data for research purposes.

## Ethical statement

The study protocol was reviewed and approved by the Institutional Ethics Committee (IEC) prior to the commencement of participant recruitment and data collection (Ref. No. **CDCRI/DEAN/ETHICSCOMMITTE/STAFF/OMR-05/2023**; approval date: **31 January 2023**). The study was conducted in accordance with the ethical principles of the Declaration of Helsinki and applicable national guidelines for biomedical and health research involving human participants.

## Author contributions

**Dr. Sravanthi Ennala:** Conceptualization, study design, methodology, clinical coordination, data collection, interpretation of implant-related clinical findings, and manuscript review.

**Dr. Gopal Srivastava:** CBCT imaging assessment, radiographic data acquisition, evaluation of bone morphology and anatomical constraints, data curation, and manuscript review.

**Dr. Aniket Anil Bonde:** Clinical data collection, intraoperative assessment of implant placement difficulty, documentation of surgical findings, data curation, and manuscript review.

**Dr. Priti P. Shah:** Supervision, study methodology, clinical oversight, interpretation of findings, critical revision of the manuscript, and final approval of the version to be published.

**Dr. Rameesa Razak:** Radiographic evaluation, CBCT data interpretation, assessment of anatomical constraints, data validation, and critical review of the manuscript.

**Dr. Mohd Zeeshan Ahmad:** Clinical data collection, study coordination, data management, interpretation of clinical findings, and critical revision of the manuscript.

**Dr. Suleiman Talha:** Implantology-related clinical assessment, evaluation of implant placement difficulty, interpretation of surgical findings, clinical validation of the study outcomes, and manuscript review.

**Dr. Nancy Jidiya:** Project administration, coordination of radiographic and clinical data collection, data validation, manuscript preparation and editing, correspondence with the journal, and final approval of the manuscript.

**Dr. Santosh R. Patil:** Statistical analysis, machine-learning methodology, data preprocessing, model development and performance evaluation, interpretation of AI-generated results, and critical revision of the manuscript.

**Dr. Shikhar Daniel:** Study conceptualization, literature review, methodological development, scientific writing, manuscript preparation, interpretation of results, critical revision of the manuscript, and overall scientific supervision.

**All authors** contributed substantially to the conception and/or design of the study, acquisition, analysis, or interpretation of data; participated in drafting or critically revising the manuscript; approved the final version for publication; and agreed to be accountable for all aspects of the work.

## Human and animal statement

All patient data were anonymized prior to analysis to maintain confidentiality. The study adhered to ethical standards for biomedical research involving human participants, and no additional radiation exposure was incurred as only pre-existing CBCT scans were utilized.

## Source(s) of support

No funding was received for this study.

## Declaration of competing interest

The authors declare that they have no known competing financial interests or personal relationships that could have appeared to influence the work reported in this paper.
